# Parasites, depredators, and limited resources as potential drivers of winter mortality of feral honeybee colonies in German forests

**DOI:** 10.1007/s00442-023-05399-6

**Published:** 2023-06-26

**Authors:** Patrick L. Kohl, Benjamin Rutschmann, Luis G. Sikora, Norbert Wimmer, Volker Zahner, Paul D’Alvise, Martin Hasselmann, Ingolf Steffan-Dewenter

**Affiliations:** 1 Department of Animal Ecology and Tropical Biology, Biocenter, University of Würzburg, Würzburg, Germany; 2 NaturKonzept, Pfullingen, Germany; 3 Bayerische Landesanstalt Für Wald Und Forstwirtschaft, Freising, Germany; 4 Forest Ecology and Management, University of Applied Sciences Weihenstephan-Triesdorf, Freising, Germany; 5 Department of Livestock Population Genomics, Institute of Animal Science, University of Hohenheim, Stuttgart, Germany; 6 Present Address: Institute for Medical Microbiology and Hygiene, University Hospital Tübingen, Tübingen, Germany

**Keywords:** Wild honeybees, Survival rates, Tree cavities, Landscape composition, Conservation

## Abstract

Wild honeybees (*Apis mellifera*) are considered extinct in most parts of Europe. The likely causes of their decline include increased parasite burden, lack of high-quality nesting sites and associated depredation pressure, and food scarcity. In Germany, feral honeybees still colonize managed forests, but their survival rate is too low to maintain viable populations. Based on colony observations collected during a monitoring study, data on parasite prevalence, experiments on nest depredation, and analyses of land cover maps, we explored whether parasite pressure, depredation or expected landscape-level food availability explain feral colony winter mortality. Considering the colony-level occurrence of 18 microparasites in the previous summer, colonies that died did not have a higher parasite burden than colonies that survived. Camera traps installed at cavity trees revealed that four woodpecker species, great tits, and pine martens act as nest depredators. In a depredator exclusion experiment, the winter survival rate of colonies in cavities with protected entrances was 50% higher than that of colonies with unmanipulated entrances. Landscapes surrounding surviving colonies contained on average 6.4 percentage points more cropland than landscapes surrounding dying colonies, with cropland being known to disproportionately provide forage for bees in our study system. We conclude that the lack of spacious but well-protected nesting cavities and the shortage of food are currently more important than parasites in limiting populations of wild-living honeybees in German forests. Increasing the density and diversity of large tree cavities and promoting bee forage plants in forests will probably promote wild-living honeybees despite parasite pressure.

**Supplementary Information:**

The online version contains supplementary material available at 10.1007/s00442-023-05399-6.

## Introduction

Europe is experiencing accelerated declines of its insect populations, calling for research to identify the drivers (Habel et al. 2019; Wagner 2020). In contrast to the negative trends that hold for many taxa, one of the ecologically and economically most important species, the western honeybee, seems to be less affected. Even in mid-latitude countries, the number of managed honeybee colonies increased during the last decade (FAO 2022), a trend aligning with the long-term growth of managed honeybee populations worldwide (Moritz and Erler 2016; Herrera 2020; Phiri et al. 2022). However, *Apis mellifera* also exists in the wild, and wild populations do not necessarily mirror managed population increases since the former can be limited by factors not relevant under human management.

As highly eusocial insects with large, perennial colonies, western honeybees are unique among the bee fauna of Europe. To survive in temperate climates with long winters, they depend on large cavities such as tree holes, rock crevices or burrows, in which to build their nests of beeswax comb and safely store honey as an energy reserve (Seeley and Morse 1976; Seeley 2010). Colonies must start rearing brood early in the year to be able to effectively exploit the first nectar flows. In spring, they rear several thousand drones and reproduce by fission. Typically, one to four daughter colonies leave the nest as swarms of up to 20,000 workers accompanied by the old queen and the first daughter queens, respectively, in search for new homes (Winston 1991; Seeley 2019). Swarms have a large dispersal range of up to 10 km depending on the availability of cavities (Lindauer 1955; Seeley and Morse 1977; Camazine et al. 1999; Kohl and Rutschmann 2018). The old colony is taken over by a young queen which soon mates with multiple foreign drones at aerial mating leks and starts egg-laying (Winston 1991; Woodgate et al. 2021). From the beginning of summer until autumn, colonies prepare for overwintering by creating large honey stores and producing long-lived winter bees (Seeley 2019). To obtain sufficient forage, colonies monitor and efficiently exploit a large area of more than 100 km^2^ around their nests (e.g., Visscher and Seeley 1982; Rutschmann et al. 2023). In temperate forests, the natural density of viable wild populations of honeybees is thought to be around one colony per km^2^ (reviewed by Seeley 2019). Today, wild-living colonies are known to outnumber managed hives in Africa and in parts of the species’ introduced range (Jaffé et al. 2010; Pirk et al. 2017; Visick and Ratnieks 2022), but it is assumed that self-sustaining populations have gone extinct in most parts of Europe (Pirk et al. 2017; Requier et al. 2019). Unfortunately, long-term data are lacking, and the drivers of wild honeybee declines have not been investigated (Kohl and Rutschmann 2018).Within the last decade, targeted censuses have revealed that wild-living colonies can still be found in various European countries (Oleksa et al. 2013; Fontana et al. 2018; Kohl and Rutschmann 2018; Browne et al. 2021; Dubaić et al. 2021; Oberreiter et al. 2021; Rutschmann et al. 2022). However, this does not prove the existence of viable populations. For example, a demographic study of wild-living honeybee populations in managed forests in Germany showed that these are far from being self-sustaining (Kohl et al. 2022b). Each spring, tree cavities are colonized by feral swarms that escaped from apiaries, but a high winter colony mortality prevents population establishment. Answering the question of why the survival of feral honeybees is currently hampered is relevant for nature conservation more generally because the diverse natural habitat requirements of honeybees (e.g., the presence of large tree cavities and the supply of floral resources) overlap with those of many other species.

It is commonly assumed that the decline of wild honeybees was caused by the ectoparasitic mite *Varroa destructor*, which invaded Europe in the 1970s (Thompson et al. 2014; Meixner et al. 2015). However, this is an indirect inference based on the experience that colonies managed in apiaries usually die within a few years when they are not treated against the parasite (Rosenkranz et al. 2010). While the mite certainly represents a threat to any population of naïve honeybees, there are indications that European wild honeybee populations were already extinct before the arrival of the new virus vector. For example, in his monograph on the bee fauna of Franconia from 1933, Stoeckhert already stated that wild honeybees have disappeared from their natural habitat. As the leading cause, he identified the lack of tree cavities in forests managed for timber (Stoeckhert 1933).

The availability of high-quality nesting cavities is probably an important limiting factor for wild honeybees under natural conditions (Ruttner 1988; Seeley 2010), and since managed forests provide much lower densities of cavities than natural forests, the lack of cavities is probably even more severe today (Remm and Lõhmus 2011; Courbaud et al. 2022). The only large tree cavities (> 10 L volume) that are regularly found in central European managed forests are those excavated by the black woodpecker (*Dryocopus martius*). However, there is a high competition for nest sites among a range of secondary cavity-nesting species (Johnson et al. 1993; Kosiński et al. 2010; Sikora et al. 2016; Zahner et al. 2017). For honeybee colonies that have successfully occupied a woodpecker cavity, competitive and/or antagonistic interactions at nest sites might, therefore, represent an additional challenge. During spring censuses of feral colonies, we regularly found pieces of beeswax comb on the forest floor beneath the cavity trees, suggesting that nest depredation had occurred. However, the question is whether cavity intruders are responsible for the bees’ death or whether they merely take over the cavities after the bees have passed away.

While parasite pressure and winter nest depredation are specific threats to honeybees, a key factor limiting bees and many other animal populations is food availability (White 2008; Scheper et al. 2014; Carvell et al. 2017; Ganser et al. 2021; Parreño et al. 2022). Nectar limitation is largely buffered under apicultural management because beekeepers provide sugar solution outside the main nectar flows, but for wild-living honeybees, gathering enough nectar and pollen to build up the worker population and the honey stores needed to survive the winter is a major challenge (Seeley 2019). The positive correlation between the probability of winter survival of wild-living colonies and the amount of flower-rich semi-natural habitat in the surroundings, as observed in an agricultural landscape in NW Spain (Rutschmann et al. 2022), suggests that food availability is an important limiting factor for wild-living honeybees. The colonies living in German forests might be especially prone to starvation since management practices have created dense forest stands dominated by a few tree species which—apart from seasonal pulses of honeydew secreted by tree-sucking insects—provide little bee forage compared to open habitats (Rutschmann et al. 2023).

The known populations of feral honeybee colonies in German forests can be used to explore whether parasite burden, nest depredation or landscape context are associated with winter survival. Under the hypothesis that parasites are currently limiting feral colony winter mortality, the prediction is that colonies that die are infested with higher numbers of parasite taxa or different parasite communities or suffer from higher colony-level parasite abundances than colonies that survive. For nest depredation to be a potential limiting factor, the prediction is that other animals enter honeybee nest cavities during winter and that colonies protected against intruders have a higher winter survival rate than colonies without protection. Knowing that major land cover types differ in the density of forage available for honeybees, the prediction of the forage limitation hypothesis is that surviving colonies are surrounded by landscapes with a higher proportion of flower-rich land cover than dying colonies. Here, we use observations of feral colony overwintering, associated data on colony-level parasite burden, nest cavity observations, depredator exclusion experiments and landscape analyses to test these predictions.

## Materials and methods

### Study regions and feral honeybee colonies

**Fig. 1:**
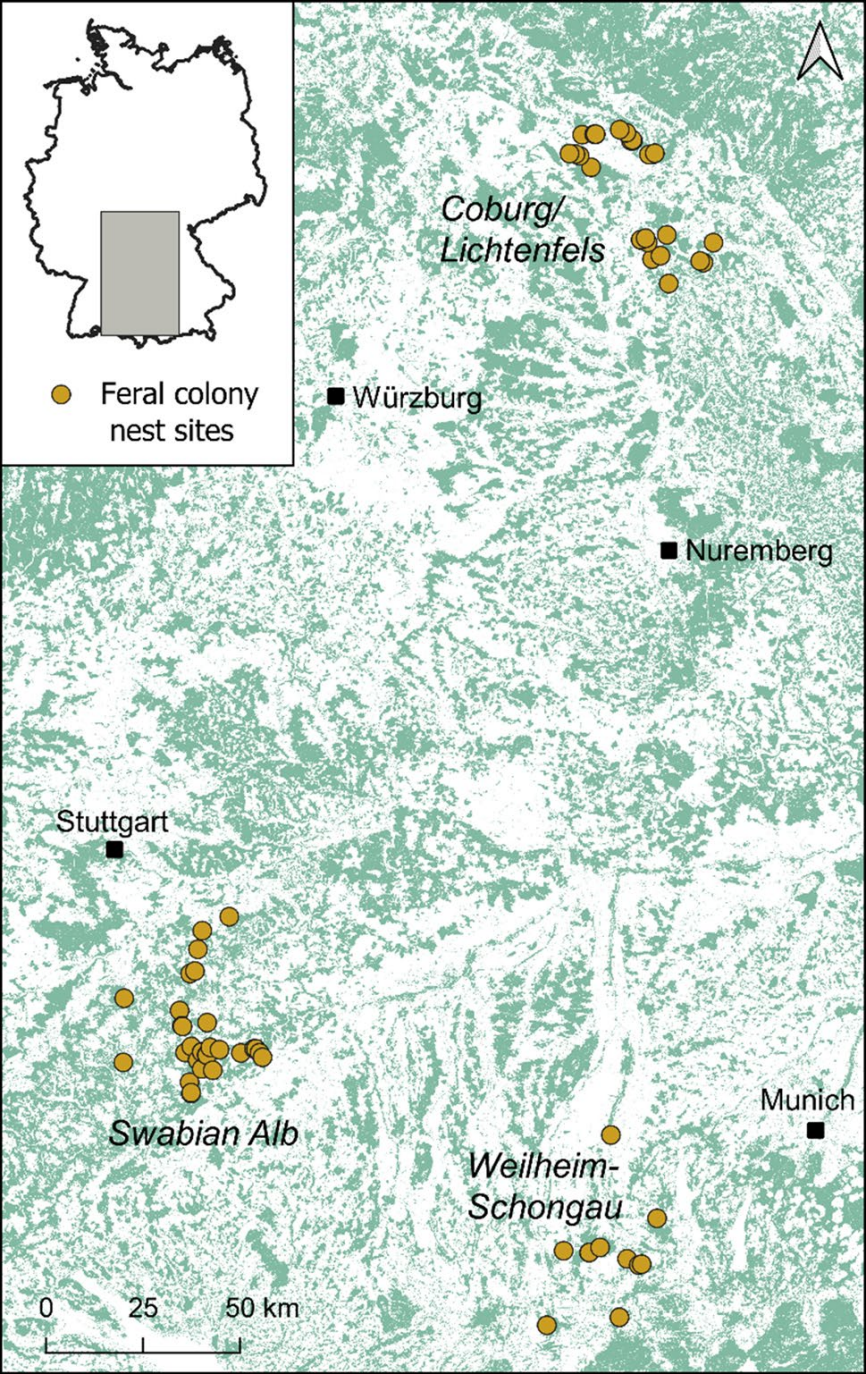
Map of the arboreal nesting sites of feral honeybee colonies considered in this study. The tree cavities (*N* = 71) were distributed over three study regions in southern Germany. Green areas denote forest based on a remotely sensed land cover map (Weigand et al. 2020). (Figure created using QGIS 3.16.8; QGIS Development Team 2021)

We considered observations of winter mortality/survival of feral honeybee colonies inhabiting managed forests dominated by beech (*Fagus sylvatica*) or spruce (*Picea abies*) in three study regions in southern Germany, each covering 500–1000 km^2^: the Swabian Alb (centre of study region: N 48.34, E 9.48), the counties Coburg and Lichtenfels (N 50.25, E 10.96), and the county Weilheim-Schongau (N 47.85, E 10.87) (Fig. 1). The survival data were gathered between 2017 and 2021 during a monitoring study that investigated the population demography of wild-living honeybees (Kohl et al. 2022b). The colonies were found by making systematic inspections of cavity trees that had been mapped before as part of regional strategies of forest nature conservation (Sikora 2009) or in connection with periodical surveys of Nature 2000 areas of the Bavarian forest department. Most colonies (> 98%) nested in cavities in beech (*Fagus sylvatica*) trees made by the black woodpecker (*Dryocopus martius*), which comprise the largest source of potential homes for honeybees in German managed forests (Kohl and Rutschmann 2018), and some colonies nested in other cavities in linden (*Tilia* spec.), spruce (*Picea abies*), or oak (*Quercus* spec.) trees. We defined “winter” as the period between late September and the beginning of April. A total of 113 colony winter survival/mortality events involving 103 unique honeybee colonies and 71 different cavities were available. Depending on the type of analysis and the availability of associated data, we either considered all overwintering observations or a subset.

### Parasite burden

To investigate whether diseases caused by parasites might be responsible for the high winter mortality of feral colonies, we tested whether there was an association between colony-level parasite burden in summer and the subsequent outcome of overwintering. We used data on the colony-level occurrence of 18 microparasites (covering eukaryotes, bacteria, and viruses) obtained using qPCR in a study comparing parasite burden between feral and managed honeybee colonies (Kohl et al. 2022a). Besides 44 colony samples that had also been used in the original study, we included data from another four colonies sampled in 2020 (these were not considered in the original study because colony age, which was a factor in the analysis, was unknown), and from 19 additional colonies collected in July 2019, totalling 67 combinations of colony-level parasite data and overwintering outcome. The parasite communities were analysed with the same method and in the same laboratory sessions as described in D’Alvise et al. (2019) and Kohl et al. (2022b). In brief, 20 bees per colony were collected at the nest entrance and total RNA was extracted from one multi-bee homogenate per colony using a TRIzol protocol. Colony-level parasite occurrence and abundance were determined from cDNA via high-throughput qPCR on a Biomark HD system (Standard BioTools, San Francisco, CA) using published primers for 18 microparasites (see supplementary information in (Kohl et al. 2022a) for a list of parasites and control genes assayed). We considered as measures of parasite burden the colony-level prevalence (presence/absence) and the colony-level loads of each parasite taxon. Parasite loads were defined as the log of the number of target molecules per 100 ng of extracted RNA.

### Nest depredation

**Fig. 2:**
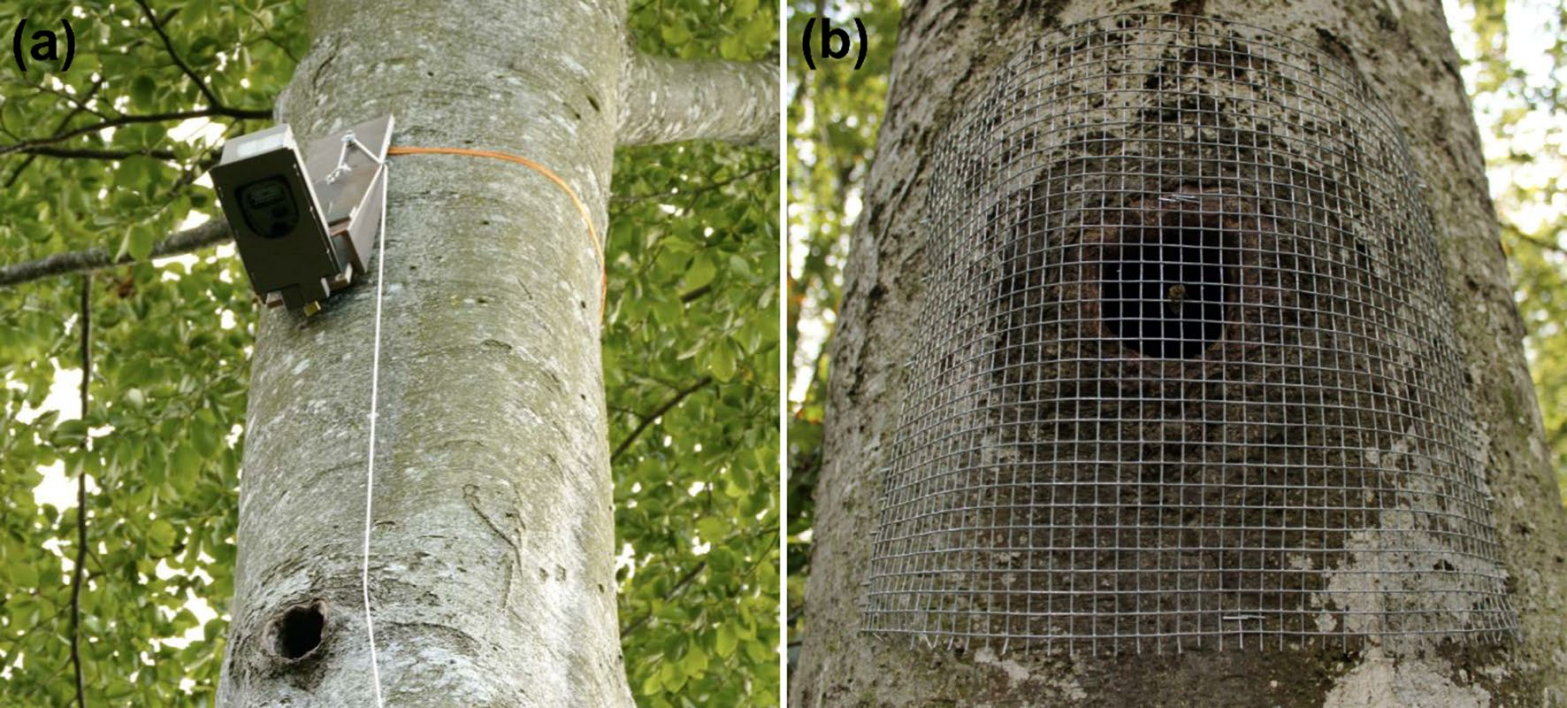
Methods to investigate nest depredation of feral honeybee colonies nesting in black woodpecker cavities. **a** Camera trap mounted approx. 1.5 m above the nest entrance. **b** Cavity nest entrance protected by wire mesh to exclude nest predators

To assess whether wild-living honeybee colonies are visited by other animals during winter and, if so, which species potentially act as nest depredators, we monitored feral colony nest entrances using camera traps (Zahner et al. 2017). A total of 15 cameras (Cuddeback Attack/Attack IR) were operated on different bee trees in 2 study regions, the Swabian Alb and the counties Coburg and Lichtenfels, between September 2019 and April 2020. We ascended trees using either a rope-climbing or a “trunk-climbing” technique (see supplementary information, Figure S1) and fixed the camera traps at 1.5–2 m above the cavity entrances using tension belts. The cameras were programmed to take one picture and one ten-second video upon motion detection, but we restricted recordings to one capture per 30 s to save battery power. A custom-built sledge system (Zahner et al. 2017) allowed us to move the cameras up and down for inspections (Fig. 2a). We checked the cameras every 6–8 weeks for data transfer from SD cards and battery changes. Due to our time-restricted recording scheme and the failures of some cameras during parts of the observation periods (due to damage by rainwater or wildlife, or quick battery exhaustion due to a high rate of false positive captures), we obtained complete records of cavity interactions for nine of the monitored cavities.

To directly test whether nest depredation negatively affects feral colony winter survival, we conducted depredator exclusion experiments. We protected cavity entrances with screens of wire mesh (mesh size: 8 mm) which we fixed to the tree trunk using a staple gun. The meshes excluded predators but allowed the passage of bees (Fig. 2b). The experiments were performed in the same two regions in which camera traps were mounted and in two subsequent winters. During winter 2019/20, we protected 12 colonies with meshes and left 20 nests open as controls, and during winter 2020/21 we had 20 mesh-protected nests and 20 control nests, totalling *N* = 32 treatments and *N* = 40 controls. Several cavity trees were considered in both the winters of 2019/20 and 2020/21. In these cases, we alternated the treatment, so the comparison was not biased by the over- or underrepresentation of any cavity.

### Landscape context

To explore whether the availability of bee forage at the landscape scale potentially limits colony winter survival, we compared the composition of the landscapes surrounding colonies that survived and colonies that died. We quantified the proportional contribution of five major land cover types (deciduous forest, coniferous forest, grassland, cropland, and settlements) to the areas within a 2 km distance of the cavity trees based on a remotely sensed land cover map (Weigand et al. 2020) using GIS software (QGIS Development Team 2021). The 2 km radii were chosen since approximately 80% of honeybee foraging takes place within this distance (Rutschmann et al. 2023) and because the landscape at the 2 km scale is known to have measurable effects on honeybee colony performance, including foraging rate, colony growth and winter survival (Steffan-Dewenter and Kuhn 2003; Sponsler and Johnson 2015; Rutschmann et al. 2022). A prior study on the spatial foraging behaviour of honeybee colonies in forest-dominated landscapes in Germany showed that the five land cover types differ in their relative value as foraging habitat, and therefore, differences in their contribution should correlate with differences in landscape-scale forage availability (Rutschmann et al. 2023).

### Statistical analyses

All statistics were performed in R (R Core Team 2022) and data figures were created using ggplot2 (Wickham 2016). We compared parasite burden between surviving and dying colonies based on three measures: the number of detected parasite taxa per colony, the community compositions of parasites, and the colony-level abundances of each assayed parasite taxon. Parasite numbers were analysed using a generalized linear model with a generalized Poisson error distribution and “winter survival” as a fixed factor (using the function “glmmTMB”, (Brooks et al. 2017)). Since we had detected regional differences in parasite numbers in the original parasite study (Kohl et al. 2022a) and since the distribution of winter survivals and deaths was not distributed equally across regions, “region” was a potential confounding factor. Therefore, we included “region” as a second predictor in the model (model formula: “glmmTMB (*Number of parasites* ~ winter survival + region, family = genpois)”). We tested for deviations from model assumptions using the functions “simulateResiduals”, “testResiduals” and “testCategorical” from the “DHARMa” package (Hartig 2022) and found none. To test the hypothesis that dying colonies had more parasite taxa than surviving colonies, we used a one-sided z-test (“glht” function from the “multcomp” package, (Hothorn et al. 2008)). To test for differences in parasite communities, we performed a distance-based redundancy analysis (dissimilarity measure: Jaccard distance) with winter survival as the constraining factor (function “dbrda” from the “vegan” package, (Oksanen et al. 2022)). Again, we factored out “region” as a potential confounding factor using the “Condition” argument (model formula: “dbrda (*Data frame of parasite prevalence* ~ winter survival + Condition (region), distance = Jaccard)”). A permutation test was used to test whether there were non-random differences in parasite communities (99,999 permutations, “anova.cca” function from the “vegan” package). We then determined, for both dying and surviving colonies, the prevalence and the mean, minimum and maximum colony-level loads of each parasite taxon, and used permutation tests to check whether there were associations between the colony-level parasite loads of any of the 18 parasite taxa and overwintering outcome (function “indepence_test” from the “coin” package, (Hothorn et al. 2016)). We used one-sided tests since the hypothesis was that dying colonies had higher parasite loads than surviving colonies.

We analysed the camera trap recordings using descriptive statistics. Based on the position of animals in relation to the cavity entrance on images and based on their behaviour as seen in the associated ten-second videos, we distinguished between cavity tree “visitations” and honeybee nest “intrusions”. Due to the time-restricted recording scheme, it was not always easy to judge whether consecutive captures were independent (different visits/different individuals). We, therefore, considered the number of camera captures (images) per species as a measure of the interaction rate with the honeybee nests. To generate an overview of relative interaction rates among different species, we considered all captures taken by the 15 camera traps regardless of whether the cameras recorded during the whole examination period. For the nine camera traps with full coverage, we created summaries of interactions as a function of time, with the number of captures binned by calendar week. We also provide the time course of average daily temperatures as obtained from two weather stations representative for the two study regions (Agrarmeteorologie Baden-Württemberg for St. Johann, www.wetter-bw.de, and Agrarmeteorologie Bayern for Birkenmoor, www.wetter-by.de). To test whether colonies protected with wire mesh had a higher winter survival rate than colonies in cavities without protection, we used a one-sided Fisher’s exact test (function “fisher.test”).

## Results

### Parasites and colony winter survival

**Fig. 3:**
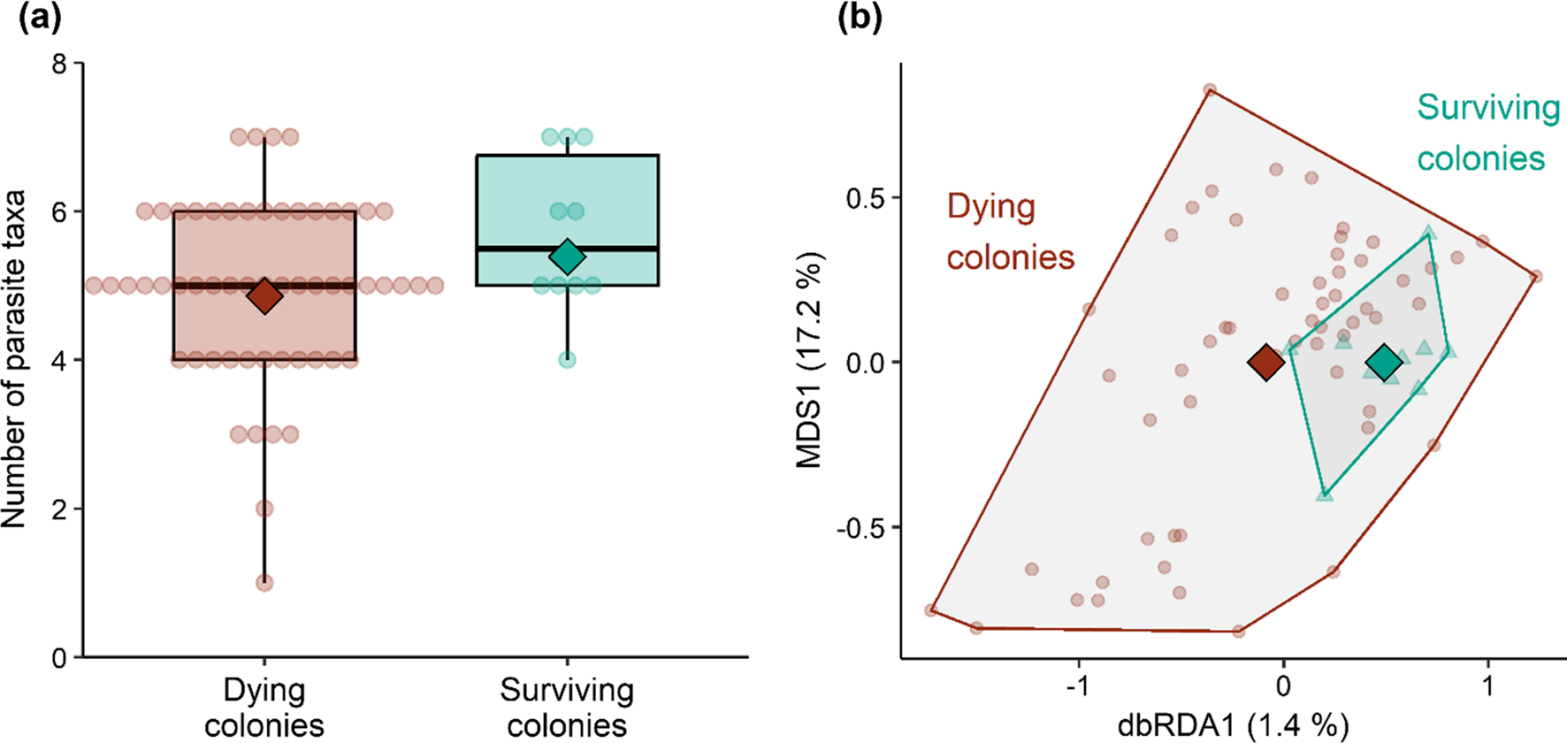
Comparison of parasite burden in dying (*N* = 57) and surviving (*N* = 10) feral honeybee colonies in the preceding summer. **a** Number of parasite taxa detected per colony among the 18 microparasites assayed. Diamonds on top of boxplots give model-estimated means, and dots are raw data. **b** Graphical representation of relative differences in parasite community composition as created by a distance-based redundancy analyses with “winter survival” as the constraining factor (effect of region partialled out). Percentages for the constrained axis (dbRDA1) and the first unconstrained axis (MDS1) give the share of explained community variation. Diamonds are means and dots (dying colonies) and triangles (surviving colonies) represent parasite communities of individual colonies

The number of parasite taxa detected per colony was not higher in the colonies that died (mean: 4.9, range: 1–7, *N* = 57) than in the colonies that survived (mean: 5.7, range: 4–7, *N* = 10) (one-sided z-test: *z* = 1.537, *P* = 0.938, Fig. 3a). The distance-based redundancy analysis revealed that parasite community compositions did not differ significantly between dying and surviving colonies (permutation test: *P* = 0.352). This is illustrated by an ordination plot in which parasite communities of surviving colonies are completely nested within the ordination space of the parasite communities of dying colonies (Fig. 3b). Looking at each microparasite in detail, we detected 13 of the 18 microparasites assayed at varying prevalences (Table 1). Dying colonies did not have significantly higher loads than surviving colonies of any of the assayed parasite taxa (one-sided permutation tests, *P* ≥ 0.18; see Table 1 for *P* values of individual tests).

**Table 1:** Comparison of prevalence and colony-level parasite loads (Log_10_[n/100 ng RNA + 1]) of 18 microparasites in D: dying colonies (*N* = 57) and S: surviving colonies (*N* = 10)

			Parasite load			
Parasite taxon	Sample	Prevalence (%)	Mean	Min	Max	*P*
*Acarapis woodi*	D	0	0	0	0	NA
	S	0	0	0	0	
*Crithidia/Lotmaria*	D	77.2	5.6	0	8.8	0.959
	S	100	7.4	6.6	8.3	
*Nosema apis*	D	8.8	0.5	0	9.4	0.188
	S	0	0	0	0	
*Nosema ceranae*	D	96.5	6.8	0	9.6	0.308
	S	100	6.3	3.3	9.4	
Bacteria						
*Melissococcus plutonius*	D	5.3	0.1	0	2.5	0.858
	S	10	0.3	0	3.2	
*Paenibacillus larvae*	D	0	0	0	0	NA
	S	0	0	0	0	
Viruses						
Acute bee paralysis virus	D	54.4	3.2	0	8.2	0.941
	S	90	5	0	7.9	
Black queen cell virus	D	94.7	5.2	0	8.3	0.517
	S	100	5.3	4.4	6.1	
Chronic bee paralysis virus	D	1.8	0	0	2.7	0.338
	S	0	0	0	0	
Deformed wing virus A	D	1.8	0.1	0	4.3	0.338
	S	0	0	0	0	
Deformed wing virus B	D	19.3	1.2	0	8.6	0.833
	S	30	2	0	7.3	
Invertebrate iridescent virus 6	D	0	0	0	0	NA
	S	0	0	0	0	
Israeli acute paralysis virus	D	3.5	0.1	0	1.6	0.820
	S	10	0.1	0	1.5	
Kashmir bee virus	D	0	0	0	0	NA
	S	0	0	0	0	
Lake Sinai virus	D	86	4.8	0	7.3	0.764
	S	90	5.4	0	7.1	
Sacbrood virus	D	45.6	1.9	0	7	0.238
	S	30	1.3	0	6.5	
Slow bee paralysis virus	D	0	0	0	0	0.992
	S	10	0.2	0	1.8	
Varroa destructor macula-like virus	D	0	0	0	0	NA
	S	0	0	0	0	

Column “*P*” gives the *P* values obtained from one-sided permutation tests of associations between parasite load and winter mortality

### Observations of cavity tree visitation and honeybee nest depredation

**Fig. 4:**
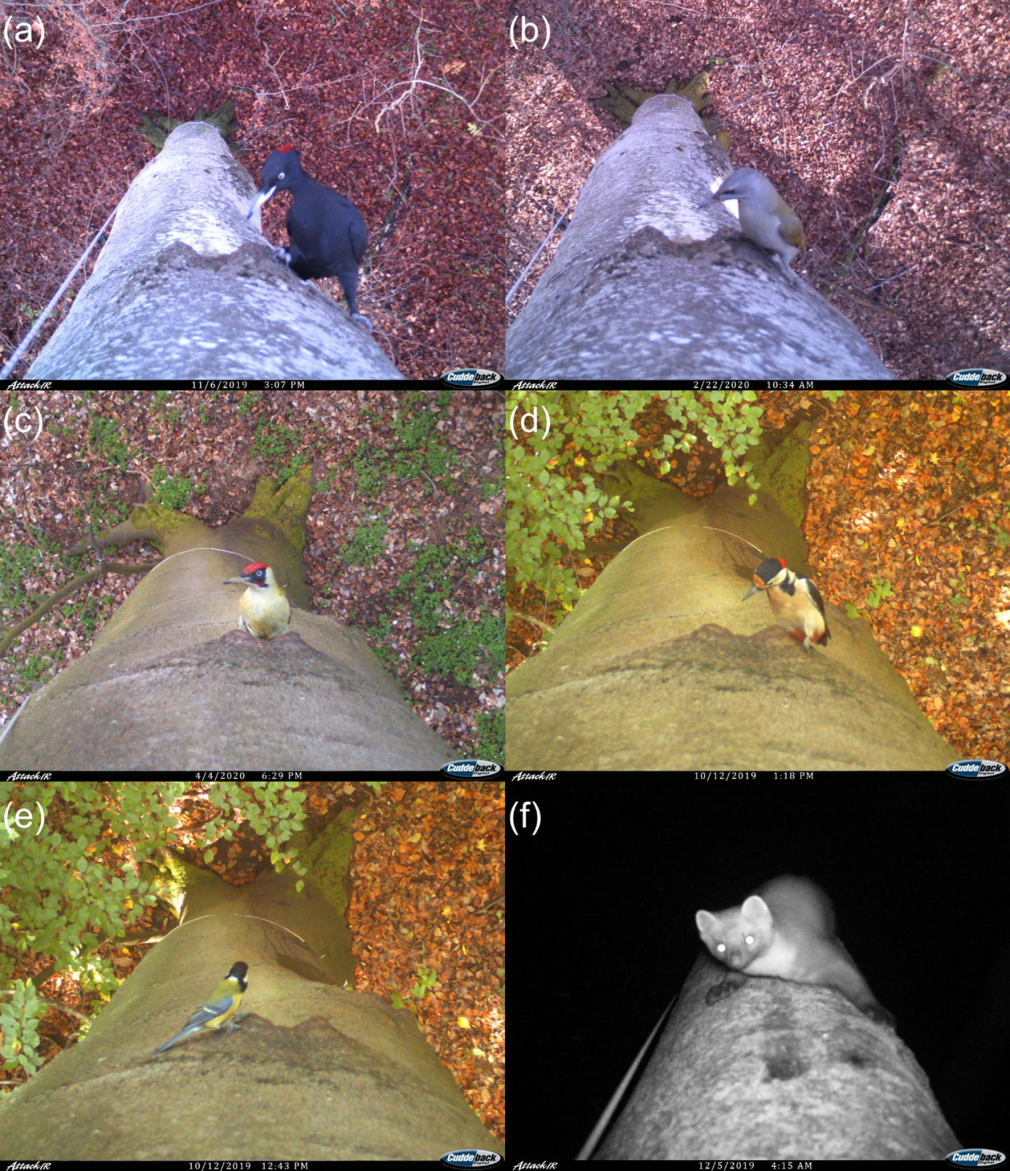
Camera trap images of six important winter visitors and depredators of honeybee nests in black woodpecker cavities. **a** Black woodpecker (*Dryocopus martius*), **b** grey-headed woodpecker (*Picus canus*), **c** green woodpecker (*Picus viridis*), **d** great spotted woodpecker (*Dendrocopus major*), **e** great tit (*Parus major*) and **f** pine marten (*Martes martes*). See supplementary information Figure S2 for images of the other visitors

**Fig. 5:**
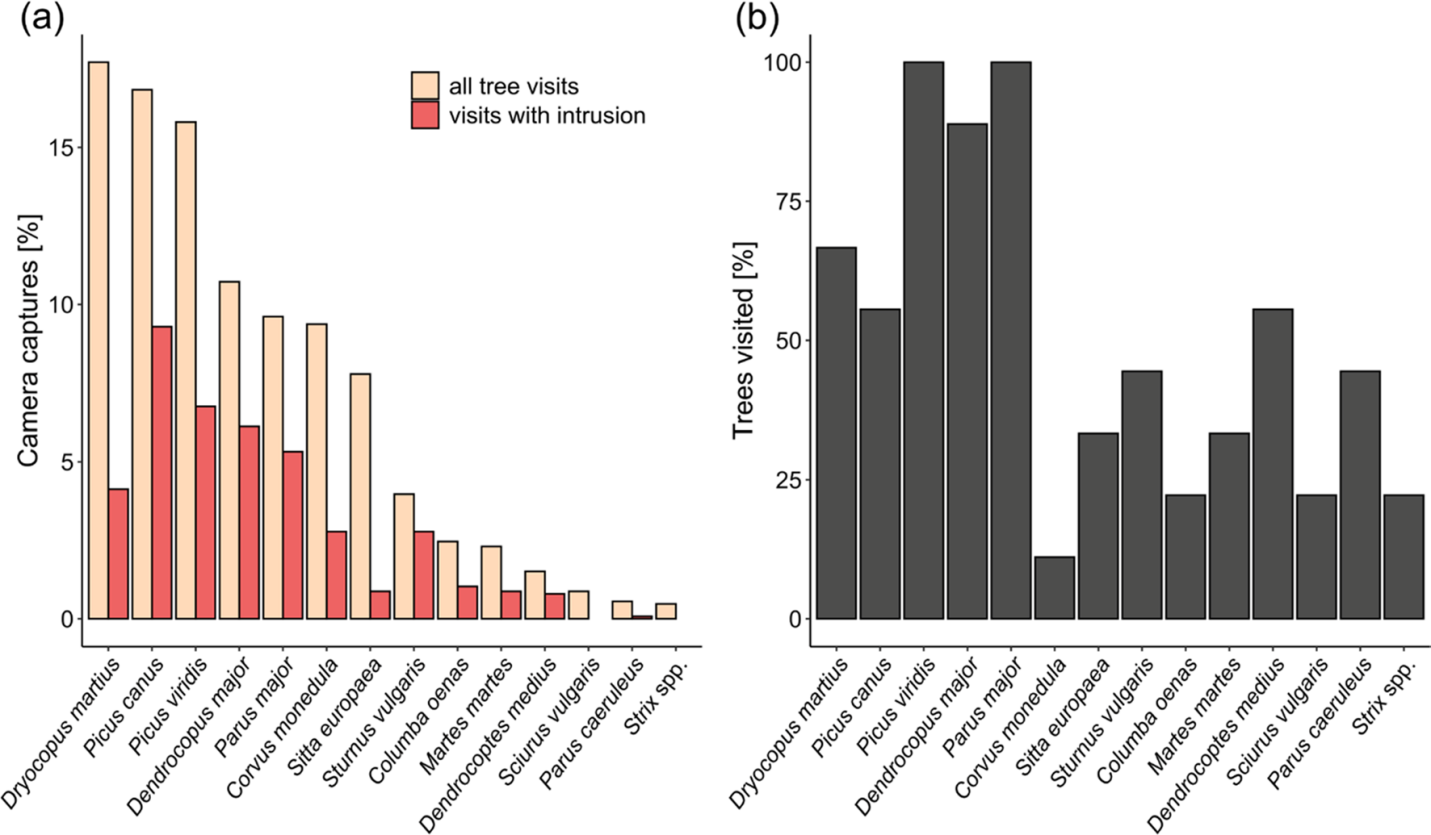
Relative contribution of 14 vertebrate taxa to bee tree visitation and honeybee nest intrusion during winter. **a** Proportion of camera captures per visitor taxon. “All tree visits” refers to all visits to the cavity trees captured by camera traps; “visits with intrusion” is a subset denoting cases in which animals entered the cavity of the bees with at least one body part. Data from all fifteen bee trees with camera traps. **b** Proportion of trees visited by each taxon. Data from nine bee trees continuously monitored with camera traps

Camera traps installed at 15 trees captured 1263 usable images between September 2019 and the beginning of May 2020, with capture frequencies ranging between 0 and 10.8 captures per tree per week. They revealed that black woodpecker cavities occupied by feral honeybee colonies are regularly visited by a range of vertebrates involving at least 13 bird species and 2 mammal species during winter (Fig. 4 and Figure S2). In 41% of the captures, visitors entered the bees’ cavities with at least one body part and thus potentially plundered the nests. The featured taxa contributed to tree visitation at different proportions and differed in their propensity to intrude into the cavities (Fig. 5).

**Fig. 6:**
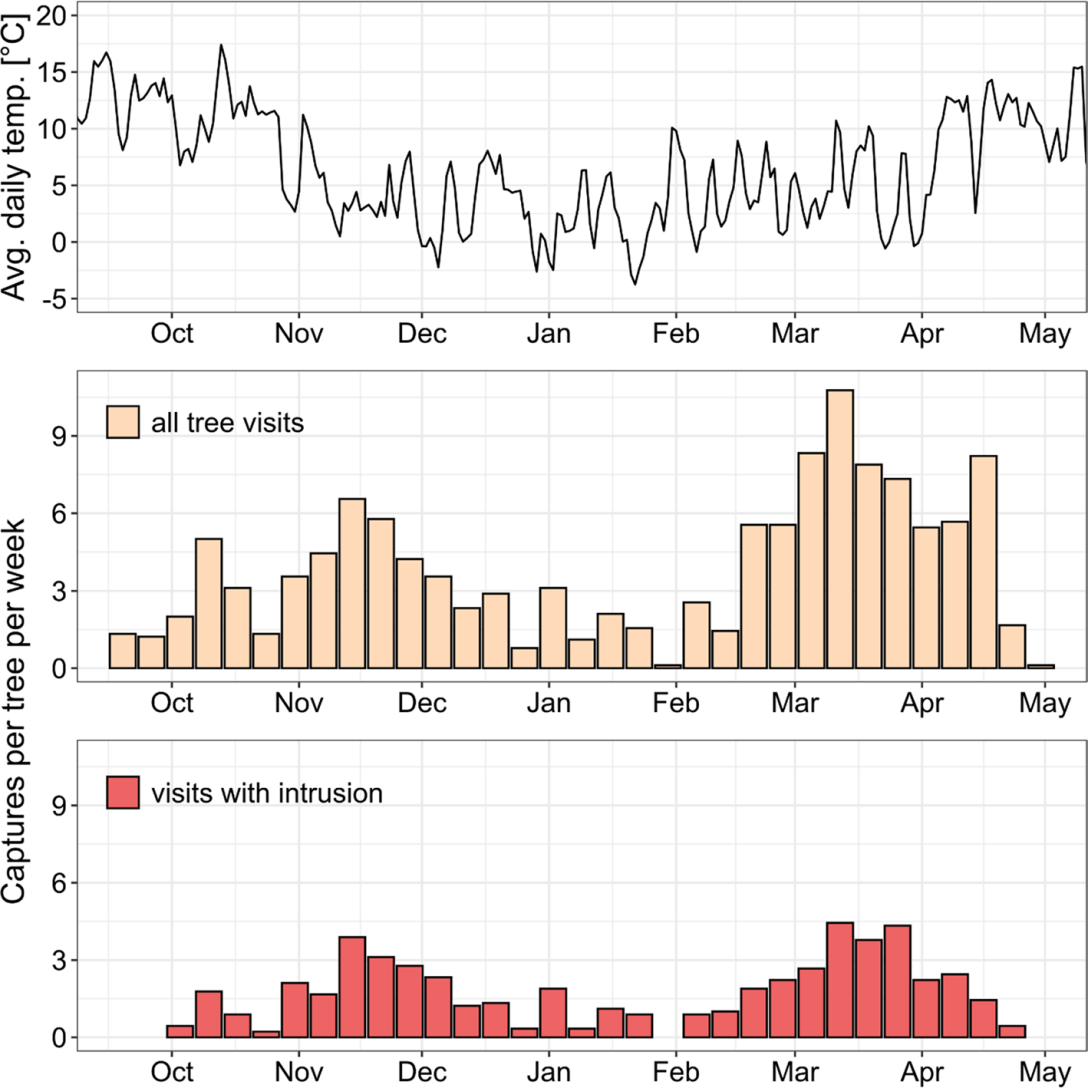
Time course of average daily temperatures and bee tree visitation frequencies by vertebrates between mid-September 2019 and early May 2020 as revealed by camera traps. Temperature data are averages obtained from two weather stations. Visitation frequencies are averaged over nine cavity trees for which we had full coverage (key as in Fig. 3)

Plotting the average frequency of camera captures as a function of time revealed a bimodal activity distribution (Fig. 6). The first peak of tree visitation in mid-November (> 6 captures per tree per week) likely mirrored an increased search for shelter in preparation for winter and/or the onset of targeted honeybee nest depredation. At the beginning of autumn, the frequency of cavity intrusion negatively correlated with temperature, indicating that potential depredators only entered cavities once the bees had ceased flight activity. The phase of relatively low tree visitation activity between mid-December and mid-February can be explained by cold temperatures and associated energy-saving behaviours of the animals. The second activity peak in March (> ten captures per tree per week) likely resulted from increased nest site search in preparation for the breeding season.

Based on the behaviour of the visitors as observed in 10-s videos (see supplementary videos at Dryad: https://doi.org/10.5061/dryad.jh9w0vtg7), the species-specific seasonal distribution of intrusion events (supplementary Figures S3 and S4), and natural history knowledge of the species’ typical breeding sites, we distinguished between depredators and species that most likely only visited cavities in search for shelter or nest sites without directly harming the bees. Six species likely preyed upon honeybees and their nests. Grey-headed woodpeckers (*Picus canus,* Fig. 4b), green woodpeckers (*Picus viridis,* Fig. 4c), great spotted woodpeckers (*Dendrocopos major,* Fig. 4d) and middle spotted woodpeckers (*Dendrocoptes medius,* Figure S2e), although not usually using black woodpecker cavities for nesting (Sikora et al. 2016), were observed sitting at the cavity entrances and pecking at combs throughout the observation period. Great tits (*Parus major,* Fig. 4e) deliberately entered the cavities of every monitored bee tree and pecked at combs from October onwards. Lastly, pine martens (*Martes martes,* Fig. 4f) were observed vigorously reaching into the honeybee nests with their forelegs or completely entering the cavities at four trees in November and February suggesting that they directly destroyed and potentially consumed honeybee nest content. Eight other species could not be clearly classified as honeybee nest depredators. The most frequent visitor species, the black woodpecker (*Dryocopus martius,* Fig. 4a), rarely entered the cavities before March. In comparison with the other woodpecker species, they showed a rather cautious exploration behaviour and rarely pecked at the bees’ combs. Blue tits (*Parus caeruleus,* Figure S2g) displayed a similar behaviour as great tits but were only rarely observed. If they are nest depredators, they do not play an important role. The other visitors (Figure S2), namely jackdaws (*Corvus monedula*), nuthatches (*Sitta europaea*), starlings (*Sturnus vulgaris*), stock doves (*Columba oenas*), red squirrels (*Sciurus vulgaris*) and owls (*Strix* spec.), are all known to use black woodpecker cavities as resting or nesting sites. Since they were either infrequent visitors or only entered and cleared cavities in spring, they were most likely searching for nest sites rather than prey.

### Effect of depredator exclusion on winter survival

**Fig. 7:**
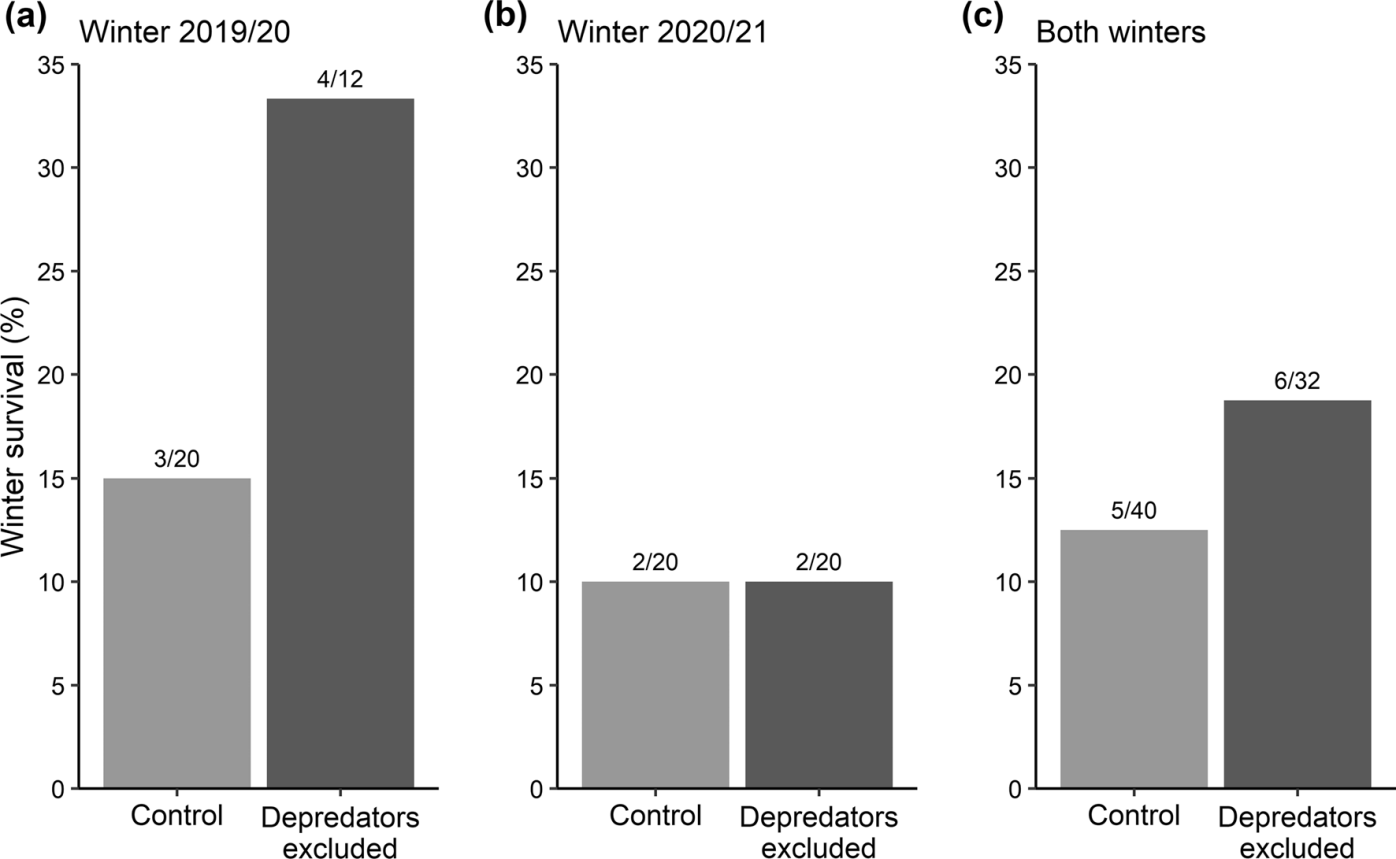
Results of the depredator exclusion experiments. Winter survival rates of colonies nesting in cavities with either open (control) or mesh-protected entrances (depredators excluded). Shown are the results for the experiments conducted in winter 2019/20 **(a)**, in winter 2020/21 **(b)**, and for both years pooled **(c)**. Numbers on top of the bars give the number of surviving colonies and the total number of cases

Honeybee colonies in nests with mesh-protected entrances had a survival rate more than twice as high (33%) as control nests (15%) in winter 2019/20 (Fig. 7a); however, the treatment and control groups had the same winter survival rate of only 10% in winter 2020/21 (Fig. 7b). Taking the results of both years together (Fig. 7c), the winter survival rate of colonies in protected nests (18.75%) was 0.5 times higher than that of unprotected colonies (12.5%), albeit this difference was not statistically significant (Fisher’s exact test, *P* = 0.342).

### Landscape context and winter survival

**Fig. 8:**
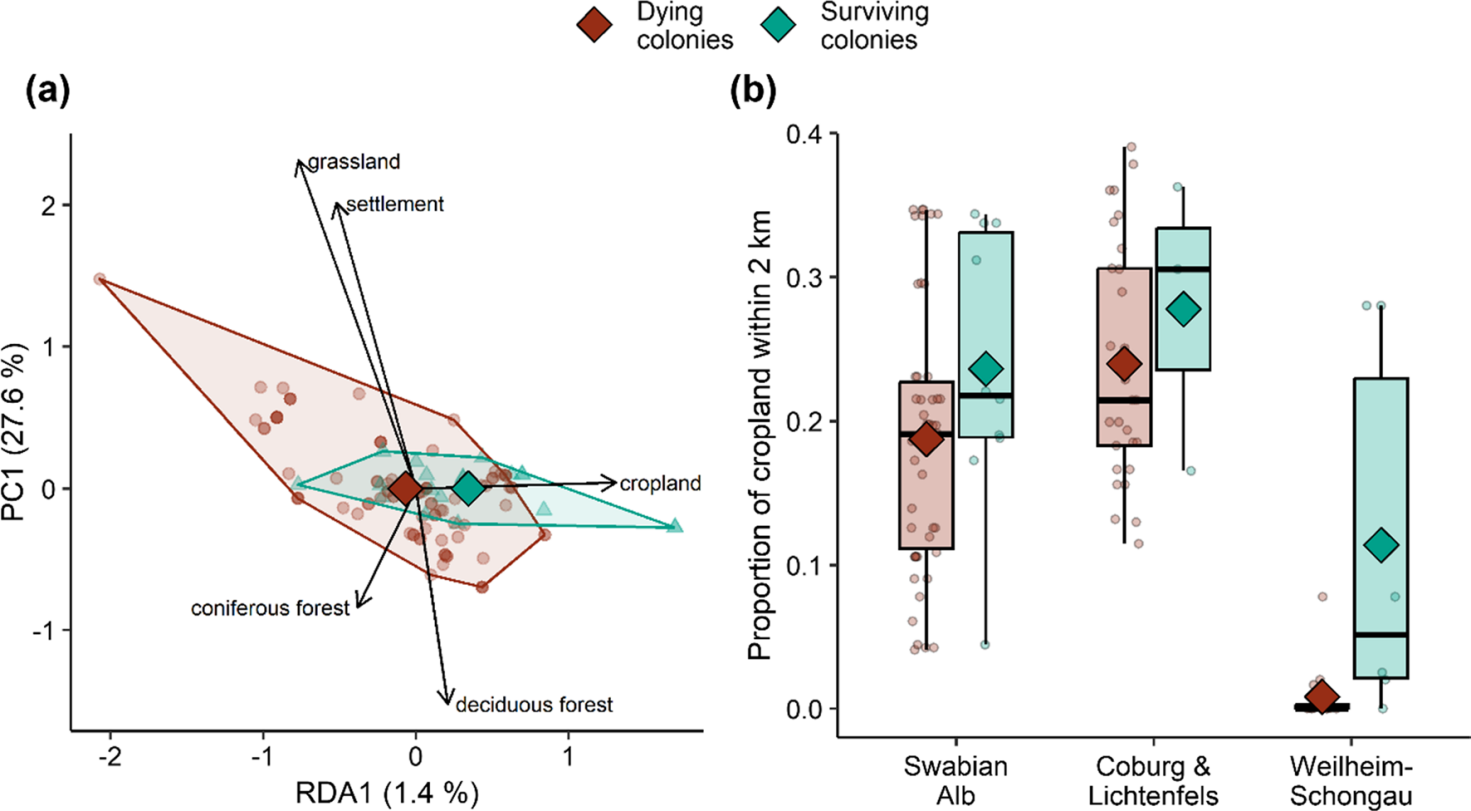
Comparison of circular landscapes (radius: 2 km) surrounding dying (*N* = 94) and surviving (*N* = 19) feral honeybee colonies. **a** Differences in the composition of landscapes as revealed by a redundancy analysis with winter survival as the constraining factor (regional differences partialled out). Percentages give the share of landscape variation explained by the constrained axis (RDA1) and the first unconstrained axis (PC1). Diamonds are means, and dots (dying colonies) and triangles (surviving colonies) represent the landscapes surrounding individual colonies. The five arrows represent the correlations of the five land cover types with the ordination axes (RDA1 is correlated with the proportion of cropland). **b** Comparison of the proportion of cropland in landscapes surrounding dying and surviving colonies for each of the three study regions. Dots are raw data and diamonds are means

A redundancy analysis revealed that the composition of the surrounding landscapes—as described by the proportions of five major land cover types within 2 km radii—differed between the nest sites of dying and surviving colonies (Fig. 8a). The difference was driven by the relative proportion of cropland and was not very likely due to chance (*P* = 0.08). Direct comparisons for each of the three study regions Swabian Alb, Coburg/Lichtenfels and Weilheim-Schongau showed that the average proportion of cropland was 4.9, 3.8 and 10.6 percentage points (mean: 6.4 points) higher in landscapes surrounding surviving colonies than in landscapes surrounding dying colonies (Fig. 8b).

## Discussion

Feral honeybee colonies populating managed forests in southern Germany have extremely low chances to survive the winter. Investigating the causes of this high winter mortality is needed for the design of effective conservation measures. Using feral colony overwintering observations gathered during a monitoring study, associated data on parasite prevalence, observations and experiments on nest depredation and landscape analyses, we made a first exploration of whether antagonistic interactions and/or landscape-level food limitations might explain winter mortality. A lack of difference in parasite burden between dying and surviving colonies suggests that winter mortality is currently not primarily caused by microparasites. Based on camera trap recordings, it seems more likely that avian and mammalian depredators are frequently involved in destroying feral honeybee colonies. Furthermore, the tendency of surviving colonies to be found in landscapes with relatively high proportions of flower-rich land cover suggests that forage availability determines winter survival chances. The hypothesis resulting from these observations is that the availability of protective nesting cavities and the provision of bee forage are currently more important than parasites in hampering feral honeybee population establishment in German forests.

Winter colony losses of managed honeybees are mainly explained by parasites (Genersch et al. 2010; Dainat et al. 2012) and, therefore, the hypothesis that increased parasite pressure is also an important factor for feral colony overwintering is well justified (Thompson et al. 2014). However, apicultural management changes honeybee ecology in several ways that make managed colonies more likely to develop high parasite loads than wild-living colonies, e.g., by producing high local colony densities (Seeley and Smith 2015; Nolan and Delaplane 2017) and by keeping large colonies in unnaturally large hives (Loftus et al. 2016). Furthermore, beekeepers support colony maintenance but prevent colony reproduction, thereby creating honeybee populations with a higher mean colony age. Since older colonies have higher parasite loads (Kohl et al. 2022a) but most wild-living colonies die at an age of less than 1 year (Kohl et al. 2022b), it needs to be scrutinized whether parasites represent a significant threat relative to other factors that kill colonies earlier in their lives. We considered here three measures of parasite burden of feral colonies and did not find any difference between colonies that died and colonies that survived the subsequent winter. The resulting conclusion is that parasites are currently not responsible for the high winter mortality. This would be incorrect if the parasite loads of feral colonies were above the thresholds typically leading to colony death, i.e., if all colonies were prone to die anyway. We think this scenario is very unlikely, given that, in our study system, the parasite burden of feral colonies was lower than that of sympatric managed colonies (Kohl et al. 2022a), and that the winter survival rate of managed colonies in Germany is generally much higher (80–96%; Genersch et al. 2010; Johannesen et al. 2022) than that of feral colonies (16%; Kohl et al. 2022a). A more serious caveat is that parasite burden in summer (we sampled colonies in July) might be a poor predictor of parasite-induced colony mortality in the subsequent winter. Quantifying parasite burden in late September or October, right before hibernation, would have been more informative (Dainat et al. 2012). However, our analyses were not unreasonable because parasite burden in summer affects the health of winter bees which are produced from August onwards (Mattila et al. 2001), and a link between parasite burden in July and winter mortality has been demonstrated before (Ravoet et al. 2013). One could argue that there were important parasites which we did not test for. For example, we did not consider the infestation levels of the ectoparasitic mite *V. destructor*, which is commonly associated with managed colony losses (Genersch et al. 2010; Dainat et al. 2012; Traynor et al. 2020). Quantifying mite abundances in feral colonies was not feasible due to limited access to bees and brood, but we think that this is not a serious limitation. On the one hand, mite infestation levels are known to highly correlate with the abundance of deformed wing viruses (DWV) (Dainat et al. 2012; Norton et al. 2021), and we tested for two common strains of DWV. On the other hand, the damaging effect of *V. destructor* is mostly due to the transmission of DWV, not due to the direct damage by the mite (Di et al. 2016; Roberts et al. 2020), which is why DWV loads are more direct indicators of honeybee health than *V. destructor* infestation levels.

The lack of evidence of an association between parasites and winter survival in present-day feral honeybee colonies casts doubt on the widespread assumption that increased parasite pressure was historically responsible for the extinction of wild honeybee populations in Europe (Thompson et al. 2014; Meixner et al. 2015). Our findings rather support the early statement of Stoeckhert that the lack of suitable nesting cavities was a major driver (Stoeckhert 1933). Today, feral honeybee colonies regularly choose old cavities of the black woodpecker as nesting sites, but this does not mean that these homes are ideal for the bees. In fact, our camera trap recordings showed that black woodpecker cavities are frequented by a range of other cavity users during winter, implying that they are generally not safe places. The black woodpecker itself, stock doves, jackdaws, nuthatches, great tits, and pine martens have previously been shown to be common users of these cavities (Kosiński et al. 2010; Sikora et al. 2016; Zahner et al. 2017). In our study, most of these species were only seen entering the bees’ cavities from March onwards, probably in preparation for the breeding season. At that time, late in winter, many honeybee colonies will have already died, so these visitors should not generally be classified as active nest-site competitors or depredators. The exceptions are great tits and pine martens. Great tits have long been known to be honeybee-eaters (Ambrose 1997). While they usually occupy less than 10% of the available woodpecker cavities during the breeding season (Sikora et al. 2016), we observed that great tits entered every single monitored cavity from October onwards, suggesting that they actively searched for the bees and preyed upon them. Pine martens were observed on four trees only, but it has been recognised before that they are true depredators of honeybees (Hood and Caron 1997). For example, Jedrzejewski et al. (1993) found that about 50% of pine marten scats contained remains of insects, with social wasps and bees (including *Apis*) making up the largest fraction, and Gunda (1968) reported that, historically, human bee-hunters in the Carpathian mountains used pine marten tracks in snow to locate bee trees, implying that the martens deliberately search for bee nests. Besides great tits and pine martens, we recorded four species which usually do not use black woodpecker cavities for nesting. Grey-headed woodpeckers, green woodpeckers, great spotted woodpeckers, and middle spotted woodpeckers clearly pecked onto the cavities and fed on the bee nest contents. While it is well known that green woodpeckers attack honeybee colonies (they also make holes in beekeeping hives) and that great spotted woodpeckers sometimes prey upon adult bees (Ambrose 1997; Floris et al. 2020), we now need to add the grey-headed woodpecker and the middle-spotted woodpecker to the list of honeybee enemies. These four woodpeckers plundered the cavities throughout the winter, suggesting that honeybees and their nests represent valuable caloric intakes for them. It was not possible to describe in detail the damage caused by the various intruders, but glimpses of the nests during the de-installation of camera traps revealed that, typically, parts of the combs were removed (supplementary information, Figure S5). However, it is still unclear whether the attacks were the actual causative factor for colony death. In the depredator exclusion experiments, honeybee colonies with protected entrances had a higher winter survival rate, but the difference to the control group with unprotected entrances was statistically not significant. However, these results are not yet conclusive. A technical flaw was that we only covered the entrances of the cavities and thus only prevented direct damage. This was unfortunate because the behaviour of the woodpeckers also involved hacking onto the outside walls of the cavities. It is known that physical disturbances of the nest can lead to colony arousal and increased winter food consumption which can be fatal when the food stores are small. Signs of recent woodpecker hacking around the entrances of mesh-protected cavities indicated that colonies that were supposed to be protected from enemies were probably also visited, and likely disturbed, by woodpeckers during winter (supplementary information, Figure S6). To properly test whether depredators affect overwintering, they need to be excluded not only from the cavity interior but from the whole tree section with the cavity. This could be achieved by wrapping the tree trunk with larger cages of wire netting. Another limitation of the experiment was the low number of replicates both in terms of mesh-treated cavities (*N* = 32) and study years (*N* = 2), and the fact that we considered the effect of nest depredation without controlling for other factors. For example, the low survival rate of only 10% among both the treatment and control groups in the winter of 2020/21 suggests that other conditions necessary for winter survival were generally not fulfilled that year, potentially masking the effect of depredator exclusion.

A factor which likely affects feral colony winter survival regardless of depredation is the abundance of food in the surroundings during spring and summer (Rutschmann et al. 2022). Honeybee colonies need about 15 kg of stored food to survive the winter in Germany (personal observations). Hoarding enough honey is especially challenging for swarms that have founded a new nest because they require extra energy to build their beeswax combs and have little time to forage until the main nectar flows are over (Seeley 2017). When considering that, among the feral honeybees colonising German forests, about 90% are recent founders (Kohl et al. 2022b), and that finding pollen and nectar is especially challenging in German forests (Rutschmann et al. 2023) a critical role of food availability seems even more obvious. We were not able to directly analyse the effect of food abundance, but the positive association between winter survival and the relative proportion of a flower-rich land cover type is an indication that landscape-scale flower availability is an important driver of feral colony winter survival. When considering the same study region, colonies that survived the winter were surrounded by more cropland. Equating cropland and honeybee foraging habitat might seem overly coarse, given that this land cover type includes fields of any cultivated plant that is seasonally harvested. However, a functional relationship between the acreage of cropland and the landscape-scale availability of bee forage is indeed plausible. Both insect-pollinated crops like oilseed rape or sunflower and wind-pollinated crops like maize disproportionately contribute to the nectar and pollen intake of honeybee colonies (Requier et al. 2015). Furthermore, an analysis of the spatial foraging patterns of honeybee colonies based on the decoding of bee dances has shown that cropland is heavily overused by the bees (Rutschmann et al. 2023).

Studying wild-living honeybees in temperate forest landscapes is extremely challenging because low population densities (often less than one colony per square kilometer) and nests high up in trees make it hard to find and access the bees (Kohl and Rutschmann 2018; Seeley 2019). We here used a unique set of colony observations to perform three independent tests of three contrasting drivers of feral colony winter mortality. Parasites, predators and food availability most likely have combined effects on colony survival (Dolezal et al. 2019), but, unfortunately, a simultaneous multi-factorial analysis was not feasible due to the limited number of colony observations and incomplete overlap in associated data. Another caveat was the naturally low ratio of survival versus mortality events, which took a toll on statistical power. Therefore, our study rather serves to redefine the likelihood of different hypotheses than to make final conclusions about the drivers of feral colony winter mortality. An important insight is that parasite burden is certainly less important than usually assumed. This is probably not because feral honeybees are not vulnerable to parasites; rather, most colonies die at a young age when they do not (yet) suffer from high parasite pressure (Kohl et al. 2022a, b). The conservation implication is that certain habitat improvements can potentially foster wild-living honeybee populations regardless of parasites. Our camera traps revealed that at least five bird species and pine martens act as depredators of honeybee nests in black woodpecker cavities and this implies that the lack of optimal nest sites is a major problem. Subject to further investigations, any action that increases the abundance of large, well-protected cavities will probably improve the abundance and winter survival chances of wild-living honeybee colonies in managed forests. Next to the cavity-related problems, it remains highly likely after this study that food limitation explains parts of the winter losses of forest-dwelling feral honeybee colonies. A promising way to further investigate the drivers of winter mortality is the use of artificial nest boxes. “Bait hives” with movable frames installed in trees have previously proven to be a valuable tool for the study of wild-living honeybees (Seeley 2007, 2017). Excluding depredators, artificially feeding the colonies, controlling mite infestation, and taking samples of bees and brood for parasite screening are all straightforward with bait hives, allowing for full factorial study designs. By installing such nest boxes not only in forests but also in agricultural and urban areas, it could further be explored whether the hierarchy of limiting factors differs between habitats. However, controlled experiments should only complement, not replace, observations of honeybees nesting in natural cavities because otherwise we miss out on, and underestimate the effect of, the diverse ecological interactions that are excluded from man-made hives.

## Supplementary Information

Below is the link to the electronic supplementary material.

Supplementary file1 (PDF 2107 KB)

## Data Availability

The data used for this publication and supplementary videos are available at Dryad (Kohl et al. 2023): 10.5061/dryad.jh9w0vtg7.
