# Supplementary material for: Parasites, depredators, and limited resources as potential drivers of winter mortality of feral honeybee colonies in German forests
Source: Oecologia. 2023 Jun 26;202(3):465–80. doi: 10.1007/s00442-023-05399-6 (PMC10386939; doi:10.1007/s00442-023-05399-6)
Supplement: Supplementary file 1 — Supplementary file1 (PDF 2107 KB) [file 442_2023_5399_MOESM1_ESM.pdf]

Supplementary information for

“Parasites, depredators, and limited resources as potential drivers of winter mortality of feral honeybee colonies in German forests”

by

Patrick L. Kohl*, Benjamin Rutschmann*, Luis G. Sikora, Norbert Wimmer, Volker Zahner, Paul D’Alvise, Martin Hasselmann & Ingolf Steffan-Dewenter

*Correspondence; email: patrick.kohl@uni-wuerzburg.de, benjamin.rutschman@uni-wuerzburg.de

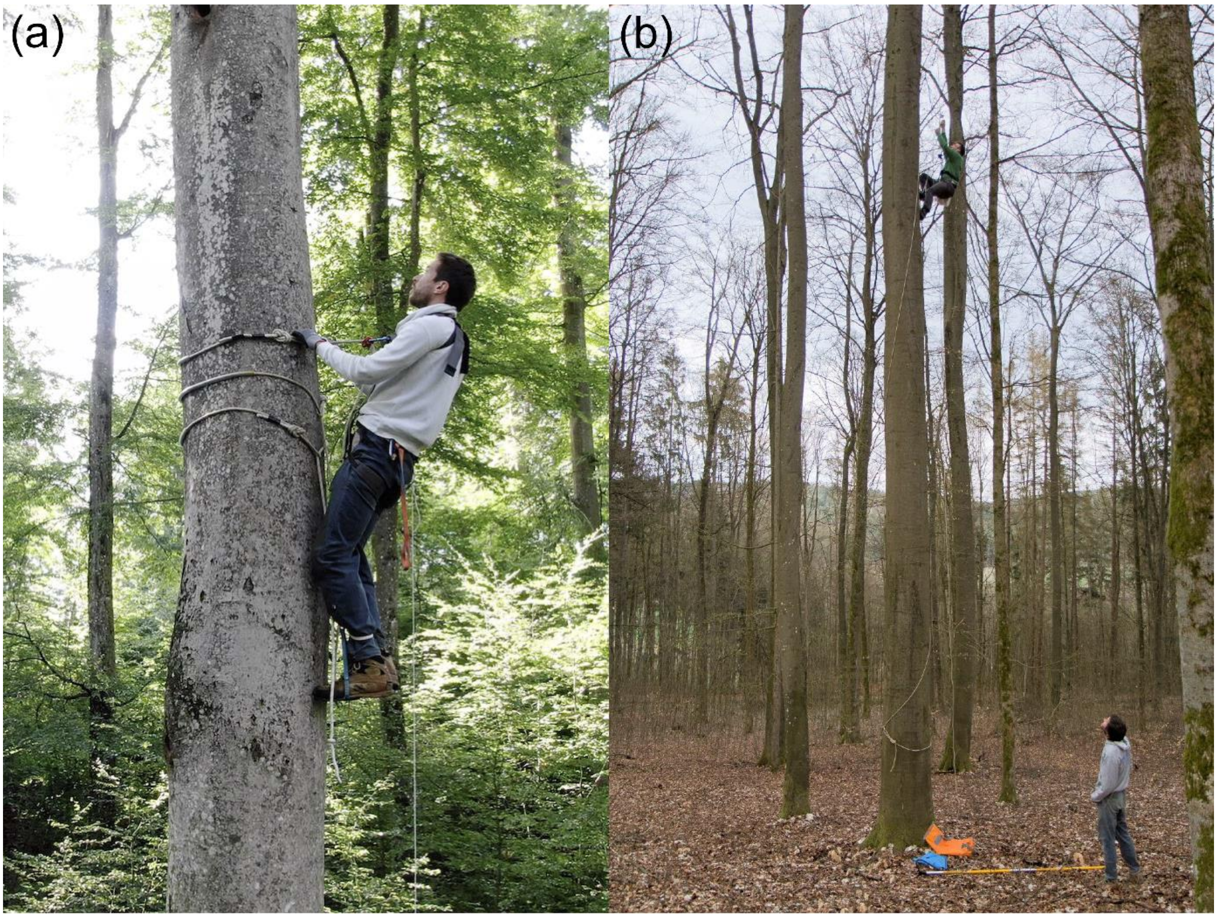

Figure S1: Methods of tree climbing used in this study. Trees were either ascended using a "trunk climbing" technique inspired by traditional tree beekeepers **(a)** or rope climbing **(b)**. For trunk climbing, three lassoes (one for the chest and one for each foot) were used to directly climb up tree trunks. For rope climbing, a semi-static climbing rope was pulled over a large branch high up in the tree and secured at the base of the tree trunk. The climbing person used a rope clamp to ascend the rope and a belay to descend. Figure (a) shows BR climbing up a tree on the Swabian Alb at the start of the experiment in 2020 (photo credit: Dimi Dumortier). Figure (b) shows PLK climbing up a tree in the county of Coburg at the end of the experiment in April 2021 (photo credit: Jean-Baptiste Pouchain).

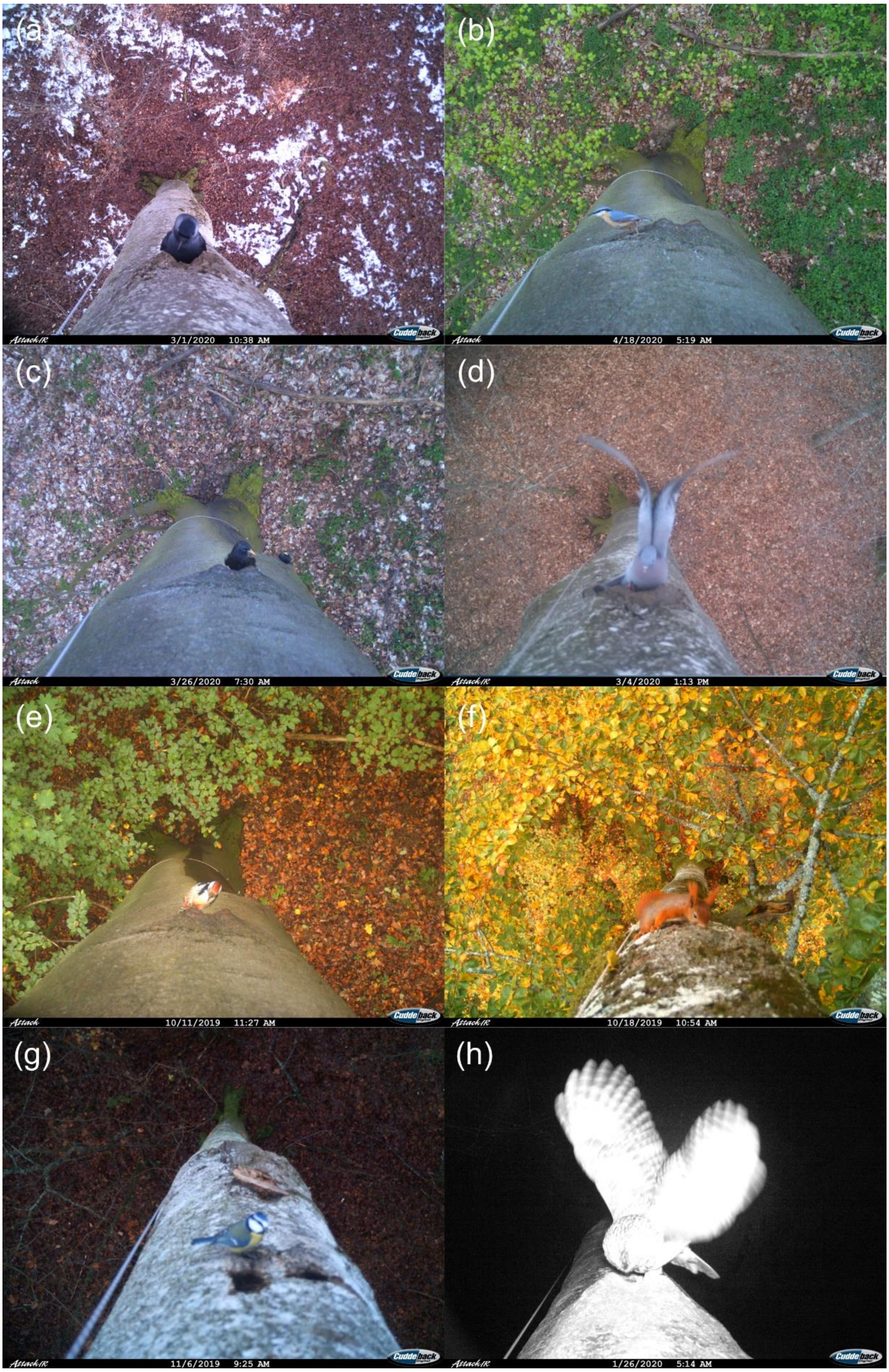

Figure S2: Camera trap images of another eight winter visitors of honeybee nests in black woodpecker cavities (see also Figure 3 in main text). **(a)** Jackdaw (*Corvus monedula*), **(b)** Eurasian nuthatch (*Sitta europaea*), **(c)** starling (*Sturnus vulgaris*), **(d)** stock dove (*Columba oenas*), **(e)** middle spotted woodpecker (*Dendrocoptes medius*), **(f)** red squirrel (*Sciurus vulgaris*), **(g)** blue tit (*Parus caeruleus*) and **(h)** an owl (*Strix* spec.).

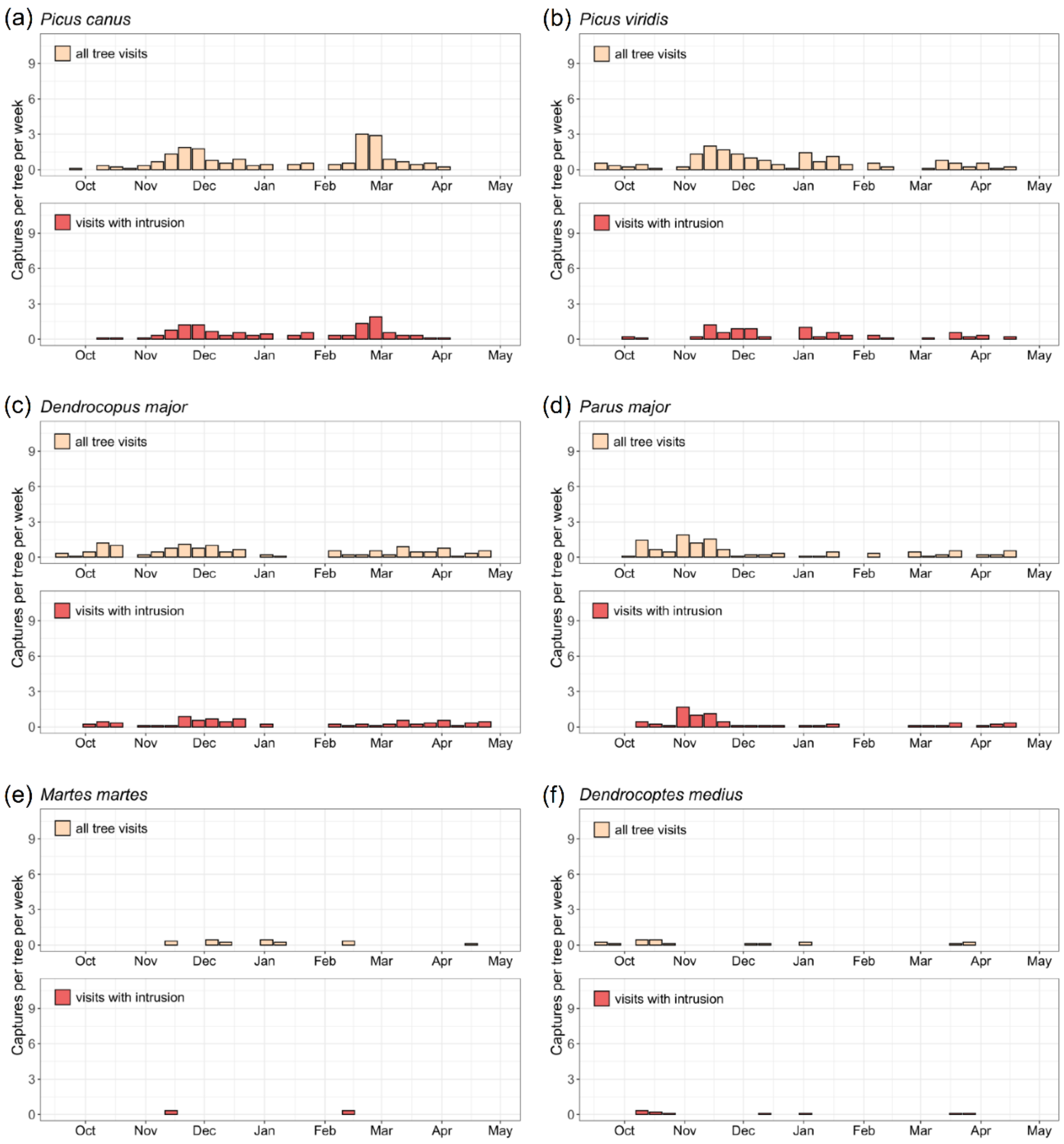

Figure S3: Time courses of bee tree visitation frequencies by different depredator species between mid-September 2019 and early May 2020 as revealed by camera traps. Visitation frequencies are averaged over nine cavity trees for which we had full coverage. The upper panels, "all tree visits", refer to all visits of the respective species to the cavity trees and the lower panels, "visits with intrusion", show a subset in which animals entered the cavity of the bees with at least one body part. Note that the behaviour of depredators is characterised by honeybee nest intrusion starting early in autumn. **(a)** Grey-headed woodpecker (*Picus canus*), **(b)** green woodpecker (*Picus viridis*), **(c)** great spotted woodpecker (*Dendrocopus major*), **(d)** great tit (*Parus major*), **(e)** pine marten (*Martes martes*), and **(f)** middle spotted woodpecker (*Dendrocoptes medius*).

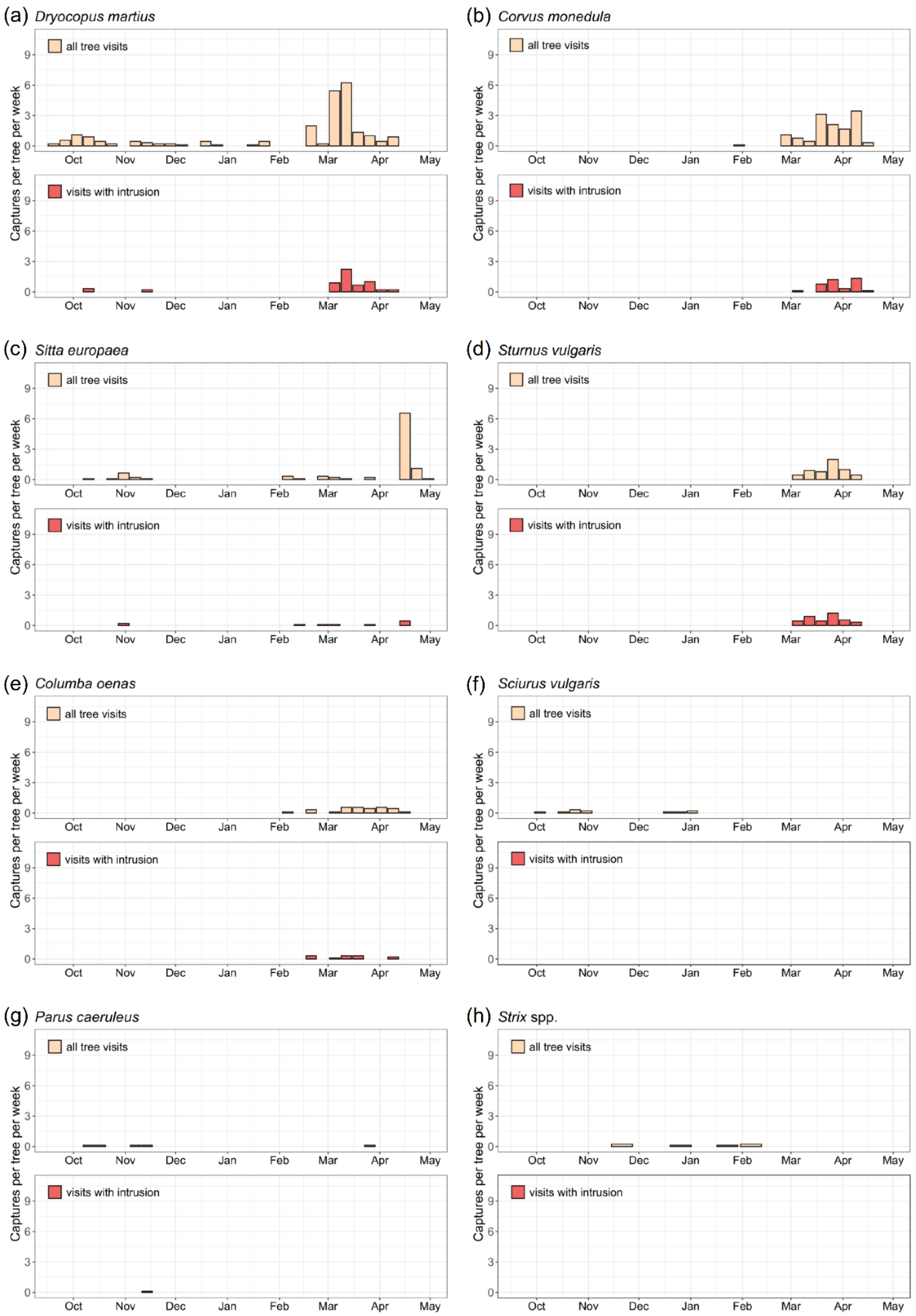

Figure S4: Time courses of bee tree visitation frequencies by different visitor species that were not classified as depredators (see Figure S2 for an explanation of the key). Note that cavity intrusions mostly happened in March, probably in preparation for breeding. **(a)** Black woodpecker (*Dryocpous martius*), **(b)** jackdaw (*Corvus monedula*), **(c)** Eurasian nuthatch (*Sitta europaea*), **(d)** starling (*Sturnus vulgaris*), **(e)** stock dove (*Columba oenas*), **(f)** red squirrel (*Sciurus vulgaris*), **(g)** blue tit (*Parus caeruleus*) and **(h)** owls (*Strix* spec.).

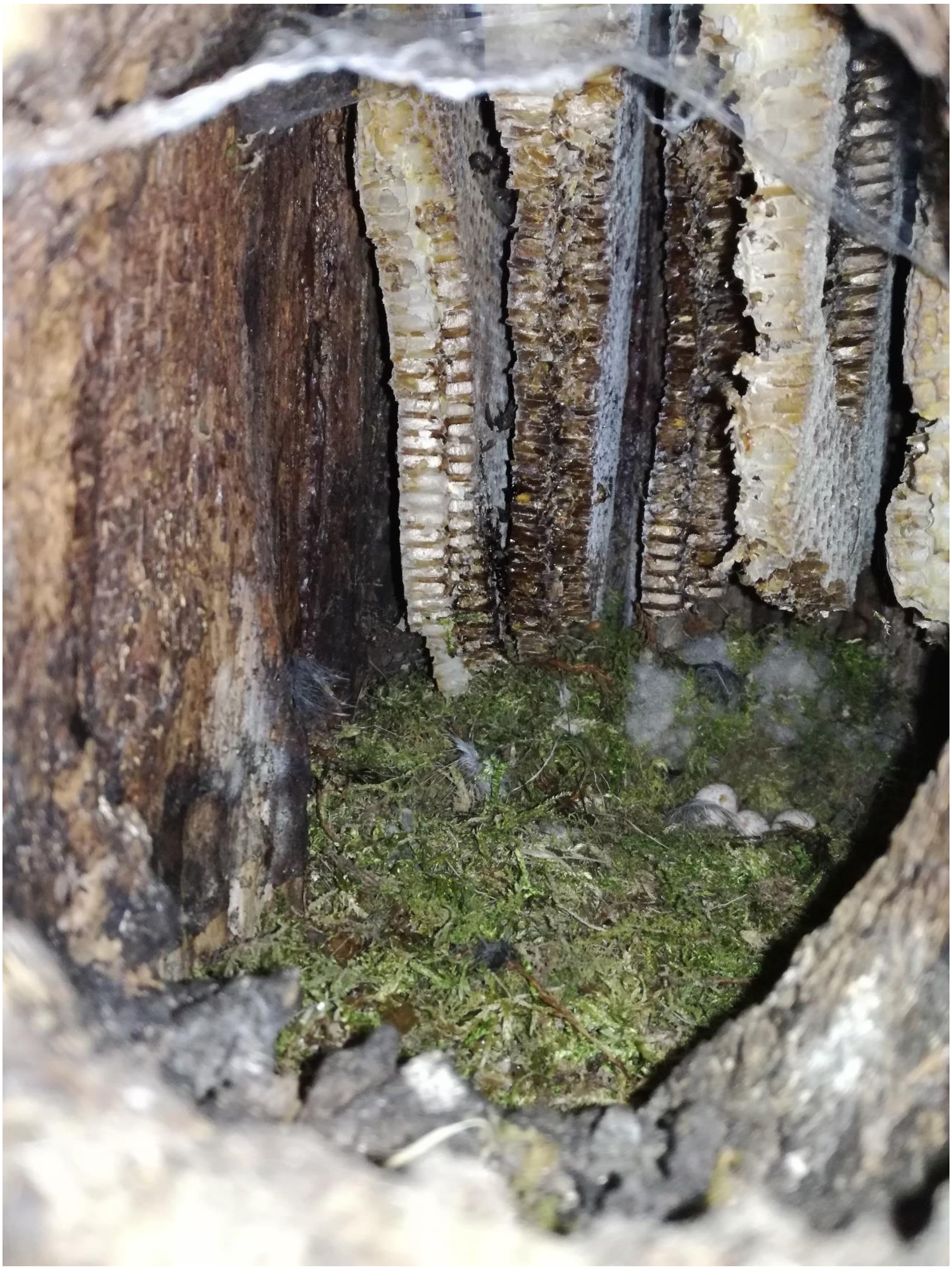

Figure S5: Image taken in April 2020 of the interior of a black woodpecker cavity that had been occupied by a honeybee colony. The bees died in winter and the cavity was taken over by a breeding pair of great tits (*Parus major*, note the clutch of eggs). The damages of the beeswax combs are typical. Photo credit: Benjamin Rutschmann.

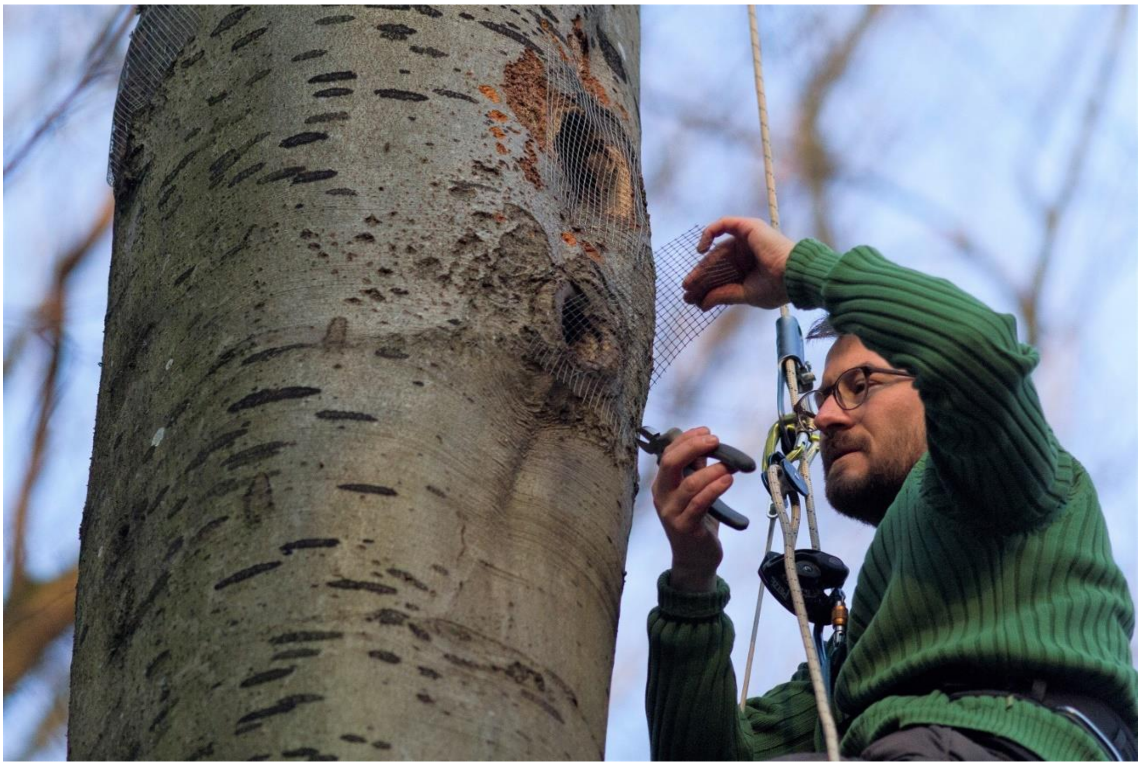

Figure S6: One of the authors (PLK) removing protection grids from a black woodpecker cavity in April 2021 (the cavity contained several entrances which needed to be sealed). Note the signs of woodpecker hacking on the margin of, and around, the main cavity entrance (the upper hole on the right side). These marks show that the treatment failed to prevent woodpeckers from working at the cavities during winter. Photo credit: Jean-Baptiste Pouchain.
